# Increased fracture rate in women with breast cancer: a review of the hidden risk

**DOI:** 10.1186/1471-2407-11-384

**Published:** 2011-08-29

**Authors:** Jean-Jacques Body

**Affiliations:** 1 CHU Brugmann, Université Libre de Bruxelles, Brussels, Belgium

**Keywords:** adjuvant therapy, aromatase inhibitor, bisphosphonate, chemotherapy-induced menopause, osteoporosis, zoledronic acid

## Abstract

**Background:**

Women with breast cancer, particularly individuals diagnosed at a relatively early age, have an increased incidence of fractures. Fractures can have serious clinical consequences including the need for major surgery, increased morbidity and mortality, increased cost of disease management, and reduced quality of life for patients. The primary cause of the increased fracture risk appears to be an accelerated decrease in bone mineral density (BMD) resulting from the loss of estrogenic signaling that occurs with most treatments for breast cancer, including aromatase inhibitors. However, factors other than BMD levels alone may influence treatment decisions to reduce fracture risk in this setting. Our purpose is to review current evidence for BMD loss and fracture risk during treatment for breast cancer and discuss pharmacologic means to reduce this risk.

**Results:**

Fracture risk during treatment for breast cancer may be influenced by the rate of BMD loss and the consequent rapid alterations in bone microarchitecture, in addition to the established fracture risk factors in postmenopausal osteoporosis. The rapid decrease in BMD during adjuvant chemoendocrine therapy for breast cancer may necessitate more aggressive pharmacotherapy than is indicated for healthy postmenopausal women who develop osteoporosis. Over the last few years, clinical trials have established the effectiveness of bisphosphonates and other antiresorptive agents to preserve BMD during adjuvant therapy for early breast cancer. In addition, some bisphosphonates (eg, zoledronic acid) may also delay disease recurrence in women with hormone-responsive tumors, thereby providing an adjuvant benefit in addition to preserving BMD and potentially preventing fractures.

**Conclusions:**

It is likely that a combined fracture risk assessment (eg, as in the WHO FRAX algorithm) will more accurately identify both women with postmenopausal osteoporosis and women with breast cancer who require bone-protective therapy.

## Review

### Incidence of Fractures in Women With Breast Cancer

Women with breast cancer (BC), even in the absence of skeletal metastases, are known to have a higher incidence of fractures than women of the same age without BC. A case control study performed before aromatase inhibitors (AIs) were part of standard medical practice showed that at the time of diagnosis, women with BC did not have a higher prevalence of vertebral fracture than controls. However, when followed after diagnosis, women with nonmetastatic BC had a higher rate of fractures compared with age- and weight-matched controls [1]. Fracture incidence was even higher (HR = 22.7; 95% CI = 9.1, 57.1; *P *< .0001) in women with recurrent disease but without skeletal metastases (Table 1) [1]. The increase in fracture incidence was maintained in analyses excluding women who eventually developed skeletal metastases (HR = 2.8; 95% CI = 1.3, 6.2). These data are rendered even more compelling by the investigators' caveat that the risk of vertebral fractures may have been underestimated because approximately 50% of the patients were taking clodronate, which has been shown to decrease rates of bone mineral density (BMD) loss and fracture [2,3]. Although it is now evident that an individual's risk of fracture can be affected by multiple health and lifestyle parameters, BMD levels and rates of BMD decrease remain key factors influencing bone health and fracture risk.

**Table 1:** Vertebral fracture incidence in women with breast cancer

	Controls^a ^(n = 776)	Breast cancer, at diagnosis (n = 352)	Breast cancer, recurrent^c ^(n = 82)
Follow-up, years ± SD	2.9 ± 0.3	2.1 ± 1.2	1.8 ± 1.4
Prevalence of vertebral fractures, %	5.2	6.0	30.5
Annual fracture incidence, %	0.53	2.72	19.21
Mean number of fractures^b^	1.08	1.45	1.69
Mean severity score^b^	2.00	2.45	4.10

Abbreviation: SD, standard deviation.

^a ^25% were current or past users of hormone-replacement therapy.

^b ^In patients with a fracture.

^c ^Excluding bone.

Adapted by permission from Macmillan Publishers Ltd: *Br J Cancer*,[1] copyright 1999.

**Figure 1:**
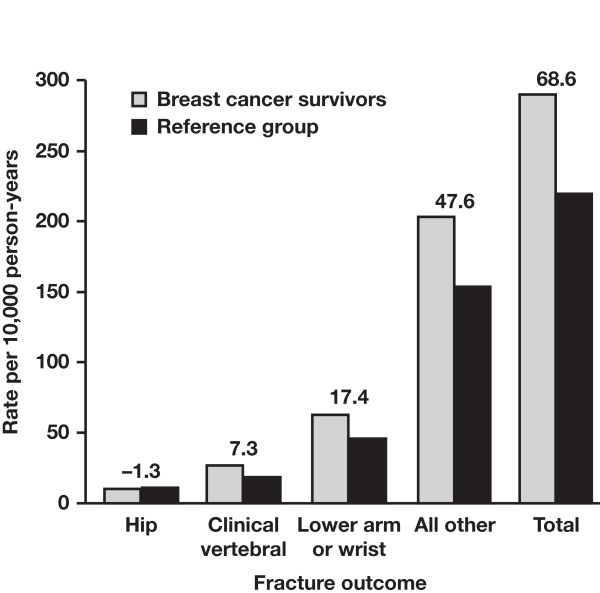
**Age-standardized fracture incidence rates**. Standardized rates were calculated using the age distribution of the entire study cohort. Excess numbers of fractures per 10,000 person-years are above each set of bars. Reprinted with permission from Chen Z, et al. *Arch Intern Med*. 2005;165:552-558 [4]. Copyright ^© ^2005, American Medical Association. All rights reserved.

In a prospective analysis of postmenopausal women from the Women's Health Initiative Observational Study (WHI-OS),[4] fracture rates for BC survivors, standardized by age, weight, ethnicity, and geographic area, were increased by 68.6 fractures per 10,000 person-years compared with women without BC (Figure 1) [4]. The increased risk of fracture was significant for women with a BC diagnosis regardless of age (HR ~1.3; *P *< .001 in the < 55 years and the ≥ 55 years groups), and was not limited to asymptomatic vertebral fractures.

The increased risk of fracture in BC patients has become even more evident following the increased use of AIs as adjuvant therapy. Despite substantial improvements in disease-free survival compared with tamoxifen, both steroidal (eg, exemestane) and non-steroidal (eg, anastrozole and letrozole) AIs have been associated with rapid loss of BMD and increased fracture risk in clinical trials [5-7]. Although this increase in fracture risk appears to be reversible on discontinuation of AI treatment,[5,6] it has now become evident that the rate of BMD loss during AI therapy far exceeds the BMD loss observed in postmenopausal osteoporosis (PMO),[8] and is therefore likely to need proactive management to preserve BMD and prevent fractures.

### Clinical Implications of Fractures

**Figure 2:**
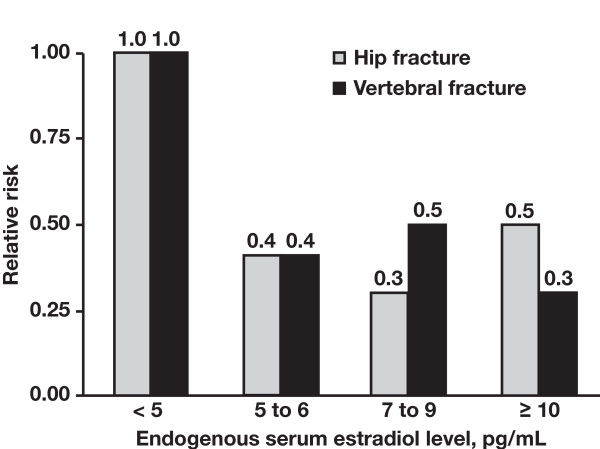
**Low estrogen levels increase relative fracture risk**. Reprinted with permission from Cummings SR, et al. *N Engl J Med*. 1998;339:733-738 [9]. Copyright ^© ^1998, Massachusetts Medical Society. All rights reserved.

The incidence of fractures increases with age because of age-related osteoporosis and decreasing estrogen levels (Figure 2) [9]. In addition, cancer therapy-induced bone loss (CTIBL) early in the course of BC, and bone metastases (malignant bone disease) in advanced disease, both contribute to increased fracture risk. Fractures and other skeletal complications can have serious clinical consequences.

Osteoporosis is characterized by decreased bone strength and increased fracture risk (including hip and vertebral fractures), and is a significant health concern in the developed world. Hip fractures secondary to osteoporosis are associated with an approximately 2-fold increase in mortality during the 12 months following the fracture [10]. Hip fractures result in prolonged hospitalization (an average of 16.3 days in an orthopedic ward and 63.6 days in a rehabilitation hospital in 1 study) and are associated with increased risks of deep vein thrombosis (DVT; reported in up to 31% of patients undergoing surgery for hip fractures), pulmonary emboli, and pressure ulcers [11-14]. Moreover, approximately 20% of patients discharged after successful surgery for hip fractures are referred back for in-patient hospital care with suspected DVT or pulmonary embolism within 3 months [12]. Vertebral fractures can be associated with chronic pain and decreased pulmonary function [15].

**Figure 3:**
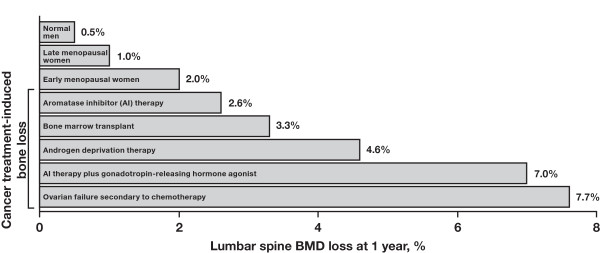
**Cancer treatment-induced bone loss occurs at higher rates than age-related bone loss**. Reprinted with permission from Guise TA [16].

Because CTIBL can occur much more rapidly than age-related PMO (Figure 3[16]), adjuvant treatment for BC substantially increases the risk of fractures, especially in postmenopausal women [17,18]. Fractures, whether attributable to PMO or to CTIBL, can significantly decrease mobility, functional autonomy, and quality of life [19]; and greatly increase disease-management costs [10]. Moreover, fractures are also associated with reduced survival (especially in the first 6 months post-fracture) [10]. Recent data indicate that all types of osteoporotic fractures increase the risk of death by 42% to 2.4-fold for at least 5 years after fracture incidents, and mortality risk remains elevated for up to 10 years after a hip fracture [20]. This article investigates the critical need for precise assessment of fracture risk to optimally manage patients with early BC.

### Risk Factors for Fracture

Numerous risk factors for the development of bone loss and fractures have been identified (Table 2) [4,21]. These are categorized as modifiable or other risk factors [4,21,22]. Some risk factors such as age are generally applicable to all women, whereas other risk factors are specific to women with BC who are receiving therapeutic interventions, including radiation therapy, chemotherapy, AIs, and steroids. For example, in women with early stage BC who were treated with conservative surgery and radiation therapy (N = 1,624), there was a dose-dependent relationship between radiation and rib fracture incidence [23]. Chemotherapy is also associated with increased fracture risk in premenopausal women with BC because it can induce early menopause and may have direct, toxic effects on bone cells. In a case-control study (N = 44 pairs), premenopausal patients with BC receiving chemotherapy had significantly lower lumbar spine BMD compared with age-matched patients not receiving chemotherapy (1.17 versus 1.29 g/cm^2^), a difference that was attributed to the high incidence of iatrogenic menopause in the chemotherapy group [24]. It has been estimated that premature menopause precipitated by adjuvant chemotherapy may hasten fractures by approximately 10 years in a large proportion of premenopausal women with BC whose disease is in remission [24]. Indeed, CTIBL in patients with chemotherapy-induced premature menopause leads to a marked decrease in BMD within a short period of time (Figure 3) [16]. In addition to chemotherapy-induced menopause, reversible ovarian suppression (eg, using goserelin) has also been associated with similarly rapid rates of BMD loss (up to 7.7% within the first year) [25].

**Table 2:** Risk factors for development of fractures or bone mineral density loss^a^

Modifiable risk factors	Other risk factors
Excessive alcohol consumption Tobacco use Existing low body mass index (< 20 kg/m^2^) and excessive weight loss Falls Sedentary lifestyle^b^ Low calcium or vitamin D intake Use of medications affecting absorption of calcium or absorption or production of vitamin D^b^ Use of corticosteroids^b^ Use of medications decreasing the production of estrogen or testosterone^b^ Low estrogen or testosterone levels	Age Low bone mass Race (Asian, white) Fracture history (personal, familial)^b^ Diabetes Rheumatoid arthritis Emphysema, chronic bronchitis Renal insufficiency

^a ^Adapted with permission of the Oncology Nursing Society from Maxwell C, Viale PH. (2005) Cancer treatment-induced bone loss in patients with breast or prostate cancer. *Oncol Nurs Forum*. 2005;32:589-603 [21].

^b ^Data from Chen Z, et al. *Arch Intern ed*. 2005;165:552-558 [4].

Aromatase inhibitors, used to suppress estrogen levels in postmenopausal women who have hormone receptor-positive BC, are also associated with rapid BMD loss and increased fracture risk. In long-term follow-up of phase III trials of AIs versus tamoxifen as adjuvant therapy for early stage BC, the incidence of fractures was reported to be 33% to 43% higher in AI-treated patients compared with tamoxifen [5-7]. This increase in fracture risk is maintained at least for the duration of AI therapy. The risk appears to wane after completion of treatment,[5,6] but more robust off-treatment data are needed to confirm this observation. Overall, because AIs are now replacing tamoxifen as the treatment of choice for postmenopausal women with early stage BC, steps should be taken to identify patients at risk for fractures to ensure proper prophylactic treatment.

Steroids (which BC patients may have received previously or concurrently for pre-existing conditions, underlying symptoms, or control of emesis) can also increase fracture risk [26]. In addition, postmenopausal women with BC may have fracture risk factors that are independent of their BC therapy, but nonetheless increase their fracture risk. Bone mineral density is an established key determinant of fracture risk and has been incorporated into many clinical guidelines for assessing fracture risk [27-29]. Osteoporosis is still defined on the basis of BMD measurement. Thus, the World Health Organization (WHO) defines osteopenia (BMD T-score < -1.0 and > -2.5) and osteoporosis (BMD T-score ≤ -2.5) relative to peak bone mass for premenopausal women and advocates bone-directed therapy for all women with osteoporosis. However, many risk factors that are independent of BMD (Table 2[4,21]) also significantly increase fracture risk, including increasing age, low body mass index, personal or family history of fractures, and current or history of smoking [22,30].

### Mechanisms of Accelerated Bone Loss in Breast Cancer Differ From Postmenopausal Osteoporosis

The risk of fragility fractures increases progressively and continuously as BMD decreases [31]. Several mechanisms contribute to bone loss in BC patients. Breast cancer itself, in the absence of bone metastases, might interfere directly with bone metabolism, for example increasing osteoclastic activity by stimulating the release of transforming growth factors [4]. Bone loss can also arise because of low estrogen levels caused by chemotherapy-induced ovarian failure or ovarian function suppression in premenopausal women [25,32]. In addition, AI therapy to suppress peripheral estrogen production in postmenopausal women can exacerbate age- and menopause-related BMD loss [5-7,17,33]. In general, decreased estrogen levels are associated with increased fracture risk (Figure 2) regardless of the mechanism underlying such hypogonadism [9].

Bone loss associated with BC treatment is substantially more rapid than during natural menopause. Women undergo an accelerated, transient phase of bone loss during natural menopause (~3% per year during the first 1-2 years, slowing to approximately 1% annually thereafter) [34]. In contrast, surgically induced menopause (oophorectomy) causes a larger reduction of total bone mass of up to 20% within 18 months in some studies, and BMD appears to continue decreasing thereafter [35]. Similarly, ovarian suppression with goserelin in premenopausal women can decrease BMD by 6% to 10% within the first 2 years [36]. The effect of goserelin-induced ovarian suppression in combination with AIs in this population is even more severe, with reports of up to 17.3% BMD loss within 3 years compared with baseline (*P *< .0001) in 1 study [25]. The rate of bone loss is also marked in postmenopausal women undergoing treatment with AIs, which block conversion of androgens to estrone and estradiol, thereby effectively eliminating estrogenic signaling [37]. This AI-associated bone loss (AIBL) continues throughout the duration of therapy, and averages approximately 2% per year [8,38]. The negative effect of estrogen depletion on bone appears to be associated with all AIs [17,33]. This class effect highlights the necessity to monitor bone loss and fracture risk in all patients receiving AI therapy, and suggests that pharmacotherapy may be needed in some patients to prevent bone loss and reduce fracture risk.

Several clinical trials have investigated bisphosphonates and other antiresorptive agents for the prevention of AIBL in nonosteoporotic patients. These include studies of oral ibandronate (Arimidex^® ^Bondronat^®^; ARIBON),[39] risedronate (Study of Anastrozole with the Bisphosphonate RisedronatE; SABRE),[40] denosumab (Hormone Ablation Therapy in Breast Cancer; HALT-BC),[41] and 4 independent trials of intravenous zoledronic acid: 1 in premenopausal (ABCSG-12)[25] and 3 in postmenopausal women (Zometa/Femara Adjuvant Synergy Trials; Z-FAST, ZO-FAST, E-ZO-FAST) [42-44]. Results from these trials demonstrate that upfront bone-directed therapy effectively prevents bone loss and maintains or increases BMD in women receiving AIs or other endocrine therapy for early BC. In addition, it has been shown that the addition of zoledronic acid to adjuvant endocrine therapy may also improve clinical outcomes (ie, delay disease recurrence in bone and other sites) compared with endocrine therapy alone in pre- and postmenopausal women with early stage hormone-responsive BC [44-46].

### Monitoring Fracture Risk

**Figure 4:**
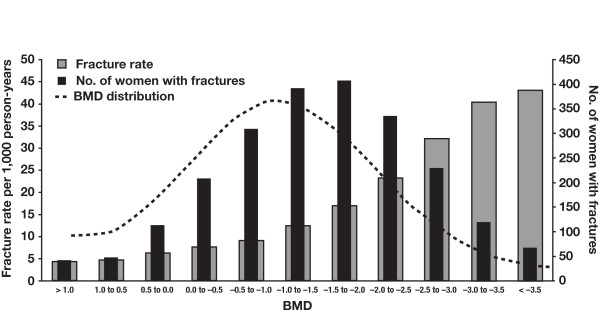
**Fracture rate, BMD distribution, and number of fractures based on NORA**. Abbreviations: BMD, bone mineral density; NORA, National Osteoporosis Risk Assessment. Reprinted with permission from Siris ES, et al. *Arch Intern Med*. 2004;164:1108-1112 [47]. Copyright ^© ^2004, American Medical Association. All rights reserved.

Although BMD is a good surrogate for bone strength, a substantial proportion of women with fractures do not have osteoporosis (defined as T-score < -2.5; Figure 4) [47]. This may be related to the fact that BMD does not capture many factors that influence bone strength, such as bone size, bone geometry, and microarchitecture change [48]. Currently, overall risk assessment, including but not limited to BMD measurements, is recommended by the WHO[29] and the National Osteoporosis Foundation (NOF),[27] which suggest using the FRAX[49] tool to compute fracture risk. The FRAX algorithm uses the femoral neck BMD T-score (if available), age, body mass index, personal and family history of fractures, corticosteroid treatment, lifestyle factors (smoking and alcohol consumption), and comorbidities (rheumatoid arthritis; secondary osteoporosis) to compute the 10-year risk of hip and other osteoporotic fractures [49]. The FRAX tool represents an important advance in understanding and accounting for the multifactorial nature of fracture risk and has been customized for various countries and ethnicities where epidemiologic data are available. The greatest benefit of the FRAX tool is the consideration of clinical risk factors for fracture, and not only BMD, in the decision to prescribe an antiosteoporotic treatment.

Estimation of fracture risk in women with breast cancer has a further level of complexity in that the disease and its treatment can, in themselves, alter BMD (and. therefore, fracture risk). The FRAX tool has some inherent limitations when applied to patients with breast cancer--because this tool has been validated using population-based studies in generally healthy postmenopausal women, it might not take factors specific to breast cancer (eg, the rate of bone loss, the effect of anticancer therapies that cause hypogonadism) into sufficient account [50]. Furthermore, FRAX does not adjust for a "dose-response" in fracture risk factors; this might be especially relevant in the breast cancer setting, wherein long-term treatment with AIs as well as other causes of bone loss would be included under the single "secondary osteoporosis" feature in the FRAX tool, thereby resulting in underestimation of the net increase in fracture risk. The duration of AI therapy is also not taken into account. Nonetheless, the FRAX index assesses fracture risk using a comprehensive list of clinical and individual risk factors, and can be used to compute long-term fracture risk even in the absence of BMD measurements [49]. Although some guidelines (eg, American Society of Clinical Oncology [ASCO] guidelines) continue to recommend thresholds for bone-directed therapy based primarily on BMD, the last 2 to 3 years have witnessed a growing awareness of fracture risk factors beyond BMD T-scores.

Recent clinical guidelines have already begun to include fracture risk factors for patient assessment and treatment decisions,[22,30,51] and have even attempted to refine the FRAX approach specifically for the breast cancer setting by using a similar combination of fracture risk factors, but with more well-defined criteria (instead of yes/no) in an attempt to address the unique bone health challenges posed by adjuvant endocrine therapy. In a recent consensus statement from an international panel of bone health experts, periodic monitoring of BMD levels is recommended for all women with breast cancer receiving AI therapy, and pharmacologic intervention is suggested for patients with normal T-scores or mild osteopenia if they experience an annual BMD decrease of 10% or more compared with pretreatment levels [52]. Moreover, this treatment algorithm recommends bone-directed therapy regardless of baseline BMD for women with multiple fracture risk factors (≥ 2 predefined fracture risk factors similar to those described in Table 2) [52]. The most comprehensive fracture risk assessment algorithm for patients with early breast cancer is described in a position statement from an expert panel in the United Kingdom [51]. This algorithm classifies patients into low-, intermediate-, and high-risk groups for fracture based on hormonal status (eg, premature menopause/use of AIs), fracture history, secondary osteoporosis, and BMD changes during adjuvant therapy for breast cancer [51]. Such algorithms for fracture risk assessment specifically in the breast cancer setting should help guide treatment decisions to preserve bone health in such patients.

## Conclusions

**Figure 5:**
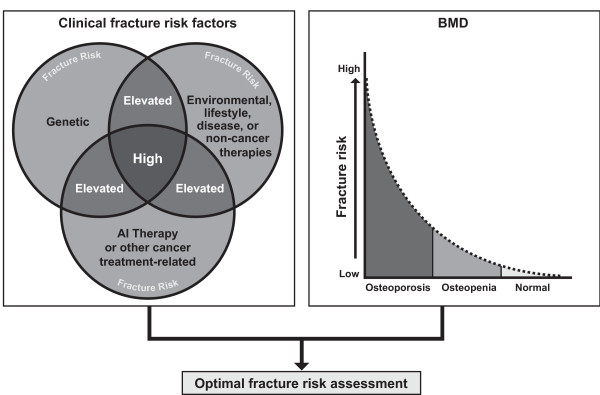
**Additive fracture risk in patients with breast cancer**. Their convergence can substantially increase risk beyond that predicted by BMD. Combining these factors with BMD provides the most accurate assessment of fracture risk. Abbreviations: AI, aromatase inhibitor; BMD, bone mineral density.

It is now clear that women with BC have an increased fracture risk compared with age-matched women without BC. In addition to established risk factors such as BMD, women with BC may be exposed to numerous factors that reduce bone strength and structural integrity. The most notable fracture risk factors include advancing age (> 65 years), AI therapy, chemotherapy-induced menopause, tamoxifen use in premenopausal women, low body-mass index (< 20 kg/m^2^), a family history of hip fracture, a personal history of fragility fracture after age 50, corticosteroid use, excessive alcohol consumption, and smoking [22,30]. The combination of these genetic, environmental, and cancer treatment-related factors contributes to the increased fracture risk observed in women with BC, especially women receiving AI therapy (Figure 5). Furthermore, a recent analysis suggests that combining clinical fracture risk factors with BMD may offer the most accurate overall assessment of fracture risk [53].

It is likely that a combined fracture risk assessment will more accurately identify both women with PMO and women with BC who require bone-protective therapy; moreover, treatment options (ie, drug choice, dose, and frequency) should probably be influenced by the severity of the BMD loss. For example, the most common therapies used to effectively treat the slow BMD loss observed in women with PMO (eg, oral bisphosphonates, calcium and vitamin D supplements) may not be optimal for prevention of accelerated BMD loss secondary to chemotherapy-induced menopause, the use of gonadotropin-releasing hormone (GnRH) analogues in premenopausal women, or AI therapy in postmenopausal women.

### Monitoring and treatment recommendations to reduce fracture risk in women with early breast cancer

Bone health assessment in women undergoing adjuvant therapy for breast cancer should include BMD T-score measurement at baseline and at least every 1 to 2 years during treatment, together with assessment of established risk factors for fracture, as defined by an international expert panel [52]. Women with moderate to severe osteopenia or additional fracture risk factors should receive bone-targeted treatment. Treatment should be continued for the duration of endocrine therapy. Currently there are no approved therapies specifically for preventing BMD loss in women receiving treatment for BC, although several recent clinical trials have sought to address this issue. The most robust data currently available in terms of the numbers of patients treated and duration of follow-up (for safety and efficacy) support the use of zoledronic acid (4 mg twice a year) to prevent CTIBL/AIBL in women receiving adjuvant endocrine therapy [42-44]. Smaller trials also support the activity of oral bisphosphonates and the new antiresorptive agent denosumab in this setting [39-41]. Although none of these trials specifically addressed the influence of clinical risk factors on fracture risk in this patient population, it is logical to infer that bone-directed therapy will be essential in women receiving AIs who also have multiple fracture risk factors. In addition, the delay in disease recurrence observed in the trials of zoledronic acid supports the potential for anticancer benefits from a therapy designed to preserve bone integrity [44-46]. Ongoing trials are evaluating whether other bisphosphonates and denosumab might also provide similar benefits,[54] and the results are eagerly awaited.

## Abbreviations

Abcsg-12: Austrian Breast And Colorectal Cancer Study Group Trial-12; AI: aromatase inhibitor; AIBL: aromatase inhibitor-associated bone loss; ASCO: American Society of Clinical Oncology; BC: breast cancer; BMD: bone mineral density; CI: confidence interval; CTIBL: cancer treatment-induced bone loss; DVT: deep vein thrombosis; GnRH: gonadotropin-releasing hormone; HALT-BC: Hormone Ablation Therapy in Breast Cancer; HR: hazard ratio; NOF: National Osteoporosis Foundation; NORA: National Osteoporosis Risk Assessment; PMO: postmenopausal osteoporosis; SABRE: Study of Anastrozole with the Bisphosphonate RisedronatE; SD: standard deviation; WHI-OS: Women's Health Initiative Observational Study; WHO: World Health Organization; Z-FAST/ZO-FAST/E-ZO-FAST: Zometa/Femara Adjuvant Synergy Trials.

## Competing interests

Dr. Body has received consultancy and lecture fees from Novartis and Amgen.

## Authors' contributions

J-JB participated fully in the conceptualization, development, review, and final approval of this manuscript.

## Pre-publication history

The pre-publication history for this paper can be accessed here:

http://www.biomedcentral.com/1471-2407/11/384/prepub
